# Peripartum takotsubo cardiomyopathy after an emergency Caesarean delivery—diagnosis and specialist management

**DOI:** 10.1016/j.bjao.2025.100478

**Published:** 2025-07-25

**Authors:** Cathriona Murphy, Deirdre Edgeworth, Gerard Giblin, Rosemarie Kearsley

**Affiliations:** 1 Department of Anaesthesiology, The Rotunda Hospital, Dublin, Ireland; 2 Department of Intensive Care Medicine and; 3 Department of Cardiology, Mater Misericordiae University Hospital, Dublin, Ireland; 4 Consultant Anaesthetist, The Rotunda Hospital, Dublin, Ireland; 5 RCSI University of Medicine and Health Sciences, Dublin, Ireland

**Keywords:** maternal critical care, maternal morbidity, point-of-care ultrasound, pregnancy, takotsubo cardiomyopathy

## Abstract

We report the case of a 32-yr-old primiparous patient who underwent an emergency Caesarean delivery and subsequently developed peripartum takotsubo cardiomyopathy necessitating transfer to a tertiary referral critical care unit and the heart failure service. Takotsubo cardiomyopathy, also called stress cardiomyopathy, is a form of left ventricular dysfunction with distinct wall motion abnormalities in the absence of coronary artery disease. It is considered extremely rare in the peripartum period, most commonly presenting in postmenopausal women with increased myocardial sensitivity to excess circulating catecholamines as a potential role in the pathogenesis. In the peripartum period, the physiological cardiovascular adaptations of pregnancy superimposed on psychosocial stressors is the assumed pathogenesis. Unexplained dyspnoea in the peripartum period should prompt early transthoracic echocardiography and specialist critical care and heart failure team input, where appropriate. This case highlights the need to consider rare cardiac causes of acute dyspnoea and demonstrates the value of point-of-care transthoracic ultrasound for prompt bedside assessment, aiding diagnosis and management. Clinical, echocardiographic, and radiological features can aid differentiating takotsubo cardiomyopathy from other differential diagnoses. Specialist multidisciplinary teams comprised of obstetricians, anaesthetists, intensivists, cardiologists, and specialist services, such as lactation and psychology services, should be available to care for these patients and a holistic approach needs to be adopted to counsel and address the psychological sequelae after such a diagnosis and appropriately plan for future pregnancies.

Takotsubo cardiomyopathy (TCM) is a form of left ventricular (LV) dysfunction in the absence of cardiac disease that is considered extremely rare in the peripartum period.^1^ Palpitations, diaphoresis, and sudden onset retrosternal chest pain are the characteristic symptoms of TCM. In the UK and Ireland cardiac disease accounts for 13% of maternal deaths.^2^ Differentiating TCM from other sinister cardiac and obstetric disease can be challenging. Although TCM is a diagnosis of exclusion and often considered a reversible disease, in the presence of acute heart failure, cardiogenic shock, or both it requires early referral to and management by specialist teams including cardiologists, specialty heart failure teams and intensivists, obstetricians, and obstetrics anaesthetists, and services such as lactation and psychology services. This case involving a healthy primiparous woman who developed TCM after emergency Caesarean delivery demonstrates the utility of point-of-care ultrasound (POCUS) in unexplained acutely deteriorating patients, early consideration of cardiac causes in previously healthy parturients, and the significance of prompt referral to specialist care. It also highlights the considerations and holistic multidisciplinary approach of caring for a critically ill mother in an adult non-obstetric intensive care unit.

## Case report

A 32-yr-old primiparous patient was admitted to a tertiary maternity hospital for induction of labour because of intrauterine growth restriction at 38 weeks and 2 days of gestation. Her background medical history was unremarkable, and she had no family history of note. She had an uncomplicated antenatal period with no evidence of hypertensive disorders of pregnancy.

The patient was transferred to the labour suite uneventfully and was managing well with Entonox (O_2_ 50%/N_2_O 50%) for analgesia with a stable cardiotocograph. She had an epidural sited successfully without complication, and she remained haemodynamically stable post insertion, receiving i.v. fluids. However, 45 min later, in the absence of maternal hypotension, a prolonged fetal deceleration occurred, which did not resolve despite adjustments to maternal position and the administration of a fluid bolus. This led to the decision to proceed with an emergency Caesarean delivery. Upon arrival in theatre the fetal heart rate recovered, and the team proceeded with a category two Caesarean delivery because of an abnormal cardiotocograph.

The epidural was supplemented successfully for delivery of the baby using lidocaine 2%, 20 ml with adrenaline 1:2000 and fentanyl 100 μg resulting in a Bromage 2 motor block bilaterally. Some 13 min after delivery of a healthy baby girl, the patient developed increasing dyspnoea (peripheral oxygen saturations between 92% and 95%) and she became progressively tachycardic (heart rate of 120 beats min^−1^) and hypertensive (180/110 mm Hg). She had received 1 L of compound sodium lactate on the labour ward before delivery and a second litre was infused intraoperatively. The patient’s dyspnoea did not improve despite facemask oxygen, and tracheal intubation became necessary. A modified rapid sequence induction with cricoid pressure was used to secure the airway. After tracheal intubation, the patient’s oxygen saturation decreased briefly to 82% and the end tidal CO_2_ remained low at 2 kPa. There was bilateral air entry on auscultation with no added sounds, peak inspiratory airway pressures were 24 cm H_2_O and on repeat laryngoscopy frothy sputum was visible at the vocal cords. The patient became hypotensive (blood pressure 90/55 mm Hg). A phenylephrine infusion was commenced to maintain cardiovascular stability. The intraoperative measured blood loss was 500 ml without the need for intraoperative cell salvage and no surgical complications were noted. After tracheal extubation, the patient was admitted to the high-dependency unit because of continued increased oxygen requirements, need for peripheral vasopressors, and invasive arterial monitoring.

Some 12 h after the delivery, the patient still required supplemental oxygen to maintain adequate oxygen saturations and was dyspnoeic, prompting a focused bedside transthoracic echocardiogram (TTE) by the obstetric anaesthesia team. This demonstrated global LV dysfunction most pronounced at the apex with an estimated ejection fraction of 20% without any appreciable regional wall motion abnormalities or LV outflow tract obstruction (LVOTO). Laboratory investigations revealed elevated inflammatory markers; a leucocytosis of 26.6×10^9^ L^−1^, a neutrophilia of 22.81×10^9^ L^−1^, C-reactive protein of 36 mgL^−1^, procalcitonin 0.19 ng ml^−1^, and a negative upper respiratory tract viral panel. Chest radiograph revealed bilateral patchy airspace infiltration which was more pronounced on the right suspicious for infective consolidation. Diuresis was achieved with an i.v. loop diuretic, and a referral was made to critical care and specialist heart failure teams in a tertiary hospital.

On arrival to the closely located tertiary referral centre, an electrocardiogram (ECG) was performed which demonstrated sinus rhythm with loss of anterior R wave, T wave inversion in leads V2, and aVL. QT and QRS intervals were normal. A formal TTE revealed normal LV cavity size and wall thickness with an estimated ejection fraction of 35–40%. Akinesia of the mid to apical LV walls was noted with hypercontractility of the basal segments. Mild mitral regurgitation (MR) was present. The LVOT Velocity Time Integral (VTI) was 15 cm. The right ventricle was normal in size and systolic function. There was no evidence of LV thrombus. Cardiac biomarkers were markedly elevated (beta natriuretic peptide 4586 ng L^−1^ and troponin 1518 ng L^−1^). CT coronary angiography confirmed coronary artery patency. The Agatston score was zero. A cardiac MRI demonstrated normal LV volumes with subtle apical dilatation and mild global hypokinesia. No oedema or abnormal myocardial enhancement were noted. Based on the clinical presentation and investigations, a diagnosis of peripartum TCM was made.

Management of acute heart failure was guided by the specialist heart failure team. An angiotensin-converting enzyme (ACE) inhibitor (captopril) and beta blocker (metoprolol) were introduced and titrated over the subsequent 72 h. Diuresis was continued. Therapeutic anticoagulation with low molecular weight heparin was commenced given the potential for development of LV thrombus. Empirical antimicrobials were commenced (piperacillin–tazobactam) for a superimposed pneumonia. Specialist peripartum pharmacy and lactation consultant services provided expert input.

At initial follow-up 6 weeks post event with the heart failure team, mild functional limitation was noted, a TTE demonstrated preserved biventricular function without residual regional wall motion abnormalities, and normal cardiac biomarkers were noted. Subsequent follow-up including a cardiac MRI has been reassuring. Reviews with the obstetric and anaesthesia teams followed by a post-ICU clinic appointment have allowed for debriefing of the events, referral to psychology services, and counselling regarding future pregnancies.

## Discussion

Takotsubo cardiomyopathy, also called stress cardiomyopathy, is a form of LV dysfunction with distinct wall motion abnormalities in the absence of coronary artery disease.^1^ Takotsubo translates from Japanese as octopus pot and describes the characteristic ballooning of the LV apex.^3^ Since it was first described in Japan in 1990, the nomenclature and diagnosis of TCM continuously evolves but it is mostly defined using the revised Mayo Clinic diagnostic criteria (Table 1).^4^, ^5^, ^6^ It most commonly presents in postmenopausal women and is considered extremely rare in the peripartum period, with the first report of peripartum TCM being described in 2008 by Muller and colleagues.^4^^,^^7^ More recently, TCM has been subdivided into primary or secondary based on the identification of an underlying inciting trigger; in the former, triggers tend to be psychological or non-identifiable, with physical or mixed triggers predominating in the latter. In secondary TCM, patients may already be hospitalised for another reason, as in our case.^4^ Takotsubo cardiomyopathy is a diagnosis of exclusion, meaning that other conditions such as peripartum cardiomyopathy, eclampsia, spontaneous coronary artery dissection, amniotic fluid embolism, hypertensive disorders or pregnancy and transfusion reactions, if relevant, must first be ruled out. An important learning point from this case, is the importance of considering acute cardiac disease in a young, healthy female with no prior medical history, as delayed diagnosis could lead to increased maternal morbidity and mortality.

**Table 1:** Mayo diagnostic criteria for the diagnosis of takotsubo cardiomyopathy.

1. Transient hypokinesis, akinesis or dyskinesis of the left ventricle mid segment with or without apical involvement. The regional wall motion abnormalities typically extend beyond a single coronary artery distribution 2. Absence of obstructive coronary disease or angiographic evidence of acute plaque rupture. 3. New ECG abnormalities (ST segment elevation, T wave inversion, or both) or modest elevation in cardiac troponin 4. Absence of • Recent significant head trauma • Intracranial bleeding • Phaeochromocytoma • Myocarditis • Hypertrophic cardiomyopathy

Peripartum myocardial dysfunction is traditionally associated with either ischaemic heart disease or peripartum cardiomyopathy, each of which requires distinct management approaches and carries different prognoses.^5^ Peripartum cardiomyopathy is a rare form of heart failure characterised by a dilated cardiomyopathy which classically presents with acute heart failure symptoms and carries a poorer prognosis compared with TCM. Takotsubo cardiomyopathy is considered a temporary heart condition (Table 2). The pathogenesis of TCM is believed to be linked to the rapid decline in oestrogen concentrations after the delivery of the placenta, which increases myocardial sensitivity to excess circulating catecholamines. Alongside the inherent physiological stresses of pregnancy, psychological triggers also play a role in the condition's aetiology. Other potential triggers include intracranial pathology and phaeochromocytoma, which can be challenging to diagnose during pregnancy as their symptoms may mimic those of preeclampsia.^4^ As in this case, the inciting trigger is presumed to be pregnancy and unanticipated emergent delivery imposing both physical and psychological stressors on the patient.

**Table 2:** Differentiating features of takotsubo cardiomyopathy *vs* peripartum cardiomyopathy. CKMB, creatine kinase myocardial band; LV, left ventricle; PPCM, peripartum cardiomyopathy; TCM, takotsubo cardiomyopathy.

	TCM	PPCM
Onset	TCM most commonly occurs within hours to days after delivery but has also been reported antenatally and in the late postpartum period.	Onset of PPCM is typically in the last month of pregnancy or within 5 months post-partum.
Aetiology	Stress plays a role in the development of TCM. Pregnancy is considered a state of heightened physiological and emotional stress. Its occurrence is also believed to be associated with the rapid decrease in oestrogen concentrations after delivery.	Unclear but likely multifactorial with physiological changes in pregnancy, genetic, hormonal, inflammatory, and autoimmune responses implicated.
Clinical features	Presents like acute coronary syndrome.	Presents with acute heart failure symptoms.
Electrocardiogram features	New onset ST segment changes or T wave inversion.	No ST segment changes.
Echocardiography features	Segmental dysfunction with apical hypokinesia causing ‘apical ballooning’.	Global LV dysfunction and hypokinesia with LV dilatation.
Prognosis	More favourable.	Less favourable.
Recovery	Within 1 month after delivery.	Delayed recovery.

Based on case series review, TCM is thought to present in the hours and days around vaginal delivery or Caesarean section.^5^^,^^6^^,^^8^ The diagnostic approach should include ECG, cardiac biomarkers, and multimodal imaging including, TTE, coronary angiography, coronary CT angiography, and cardiac MRI. Takotsubo cardiomyopathy often mimics acute coronary syndrome and, as in this case, is generally accompanied by new ST segment changes or T wave inversion. It is estimated that currently 2% of patients undergoing emergency coronary angiography for suspected acute coronary syndrome have underlying TCM.^4^ On TTE, TCM tends to have segmental dysfunction with apical dyskinesia. In general, TCM tends to have a favourable prognosis with normalisation of LV systolic function within 6 months reported in a case series of peripartum cases.^5^^,^^6^^,^^8^^,^^9^ In this case, point-of-care echocardiography was instrumental in identifying the underlying cardiac cause of respiratory distress and escalating management to the relevant service, underlining the importance of skill acquisition in POCUS to aid patient assessment. POCUS has been well established in the critical care setting to complement bedside clinical examination. On the labour ward, where an ultrasound with a curvilinear probe is generally readily available, the addition of a cardiac phased array probe is crucial to facilitate the rapid assessment of the ill parturient while awaiting additional diagnostics and imaging.

Primary management of peripartum TCM should focus on identifying and alleviating stressors. In cases complicated by heart failure and shock, as in this case, intensive management may be necessary. Active management should involve assessing for acute coronary syndrome as an alternative diagnosis and initiating acute heart failure treatment, including ACE inhibitors or angiotensin receptor blockers, beta-blockers, and diuretics to reduce ventricular filling pressures. In the event of cardiovascular collapse, TTE can distinguish the presence or absence of LVOTO, and a chest radiograph or POCUS can help identify the presence of pulmonary oedema. Although absent in this case, LVOTO is not infrequent in cases of TCM, being caused by hypercontractility of the left basal segments resulting in systolic anterior motion (SAM) of the mitral valve, often accompanied by MR, causing narrowing of the LVOT.^10^ This distinction is important as standard inotropic treatment for shock may even be fatal in a case of TCM with LVOTO, worsening the SAM and MR exacerbating the obstruction. Vasopressors acting via catecholamine-related pathways may worsen TCM by exaggerating the myocardial stunning. Vasopressors, for example phenylephrine, which act by increasing systemic vascular resistance and decreasing heart rate, may benefit SAM and LVOTO. Furthermore, vasopressors that increase systemic vascular resistance via other pathways, such as vasopressin, should also be considered. Diuretics and nitrates are also counterproductive in cases of LVOTO as they reduce preload and afterload. Takotsubo cardiomyopathy with LVOTO should be managed as hypertrophic cardiomyopathy, with goal-directed fluid therapy and beta blockade, to reduce hypercontractility, prolong diastole, and increase end diastolic volume. Pregnancy and the peripartum period are hypercoagulable states. Both the American Heart Association and the European Society of Cardiology recommend therapeutic anticoagulation for patients with TCM who have an identified LV thrombus.^11^, ^12^, ^13^ Furthermore, in cases of extensive segmental akinesia or atrial fibrillation, therapeutic anticoagulation is advised.^11^, ^12^, ^13^ European guidelines emphasise confirming thrombus resolution and LV function recovery before discontinuing anticoagulation.^11^^,^^12^ The role of prophylactic anticoagulation remains uncertain but may be considered to prevent LV thrombus formation.^11^, ^12^, ^13^ In cases where the LV ejection fraction is <30%, apical ballooning is present, and cardiac enzymes are elevated, prophylactic anticoagulation should be considered.^14^

In addition to the intensive medical management of the cardiomyopathy; a key learning point from this case is the holistic approach and adjustments that need to be used in adult intensive care to cater to the critically ill mother, hospitalised in a non-obstetric unit. Breastfeeding support is a key part of caring for postpartum women, where initiation and maintenance of breastfeeding can be challenging. The use of lactation consultants and specialised pharmacists in this case were instrumental to the positive outcome for this patient, highlighting the benefit of early multidisciplinary involvement to advise on safety of newly introduced medicines and to support the new mother in these demanding circumstances.

This case highlights a key challenge in managing critically ill mothers: determining the most appropriate location for their treatment. The creation of a dedicated ICU for critically ill mothers remains a subject of ongoing debate within the maternal critical care community, presenting both benefits and challenges. A designated maternal ICU offers specialised care through a multidisciplinary team, including obstetricians, intensivists, anaesthetists, and neonatologists, within an optimised environment equipped with tailored protocols for the provision of specialised maternal- and family-centred care. However, implementing such a unit can be resource-intensive and logistically complex. Additionally, maternal critical care admissions are relatively rare, raising concerns about maintenance of the necessary expertise in such a unit and its cost-effectiveness for healthcare systems. Furthermore, maternal critical care admissions comprise a combination of both obstetric-specific pathologies, and conditions that require the broader expertise and resources of a general ICU.^15^ A potentially more viable alternative is the adoption of a hybrid model, which includes high-dependency facilities within obstetric units, ensuring access to maternal critical care teams, and the capacity for admission to general ICUs when necessary. However, the need for a maternal ICU is ultimately determined by the availability of resources, the structure of local healthcare systems, and the volume of cases.

There are only a few documented cases of women with a history of peripartum TCM in the literature, leaving significant uncertainties about their management and follow-up during future pregnancies.^16^ The estimated recurrence rate of TCM in subsequent pregnancies is estimated to be between 1% and 11%.^16^ Currently, there are no consensus guidelines regarding the optimal mode or timing of delivery or the most suitable type of anaesthesia for these women. However, given the role of sympathetic activity and myocardial sensitivity to catecholamines in the condition’s pathogenesis, epidural anaesthesia is considered advantageous. This allows for a more gradual onset of blockade, promoting greater haemodynamic stability and offering peripartum analgesia to prevent sympathetic surges. Additionally, it avoids the intense sympatholysis and hypotension associated with subarachnoid block. Although considered a reversible heart failure syndrome and therefore benign, a key learning point is that subsequent pregnancies should not be discouraged for women who develop peripartum TCM. These women should be informed of the risk of recurrence in subsequent pregnancies and should be identified early and closely monitored by a multidisciplinary team, including obstetricians, cardiologists, and anaesthetists. Regular cardiological follow-up is essential, with transthoracic echocardiography to assess LV function and haemodynamic status during pregnancy and postpartum.^17^ Special attention should be paid to identifying and avoiding any potential stressors that could trigger complications.

Furthermore, in the instance of a traumatic delivery, women can develop childbirth-related posttraumatic stress disorder (CB-PTSD) and so proactive referral and engagement with early psychological therapies can mitigate this negative sequela. CB-PTSD results in a significant burden of disease for women as they experience intrusive memories and flashbacks, emotional numbness, and exhibit avoidance behaviours.^18^ It can result in significant consequences leading to long-term psychological distress disincentivising future pregnancies, and affecting their physical health contributing to sleep disturbances and chronic pain. Often CB-PTSD is highly co-morbid with postpartum depression and if left untreated it can significantly impair maternal functioning in the postpartum period. This includes higher rates of breastfeeding difficulties, reduced parental confidence, strain on partner relationships, diminished maternal affection, and disruptions in maternal–infant bonding. It can result in consequences for the infant, increasing the risk of emotional, social, and behavioural problems. In this case, the patient underwent extensive multidisciplinary debriefing and engaged in cognitive behaviour therapies in addition to the clinical follow-up. This case highlights the need to consider the psychological implications of a peripartum event and holistically address the patient’s condition providing reassurance of the implications for future pregnancies.

In conclusion, peripartum TCM is a rare but potentially under-recognised cause of dyspnoea in the peripartum period and so it is important to maintain a high index of clinical suspicion to prompt early diagnosis and management. The use of point-of-care echocardiography should become mainstream in an obstetric setting and can be instrumental in assisting in diagnosis of the undifferentiated critically unwell obstetric patient. A multidisciplinary team, comprising obstetricians, anaesthetists, intensivists, cardiologists, and specialist services such as lactation and psychology services, is essential to manage a critically ill mother and ensure the best possible outcomes for patients.

## Authors’ contributions

Compilation of the first draft: CM

Liaison with fellow authors: CM, RK, DE

Obtainment of patient consent: DE

Review of manuscript, from first to final drafts, providing suggestions to improve the text: all authors

Review of manuscript from intensive care medicine viewpoint, providing invaluable content to aid editing: DE

Review of manuscript from cardiology viewpoint, providing invaluable content to aid editing: GG

Editing various drafts of the manuscript: CM, RK, DE

Provided images: DE

## Declarations of interest

The authors declare that they have no conflicts of interest.
